# Assessing the impact of dietary habits on health-related quality of life requires contextual measurement tools

**DOI:** 10.3389/fphar.2015.00101

**Published:** 2015-05-08

**Authors:** Cristina Ruano-Rodríguez, Lluis Serra-Majem, Dominique Dubois

**Affiliations:** ^1^Nutrition Research Group, Research Institute of Biomedical and Health Sciences, University of Las Palmas de Gran Canaria, Las Palmas de Gran Canaria, Spain; ^2^Ciber Fisiopatología Obesidad y Nutrición, Instituto de Salud Carlos III, Madrid, Spain; ^3^Pharmed, Université Libre de Bruxelles, Brussels, Belgium

**Keywords:** dietary habits, quality of life, health outcome measures, general population, review

## Abstract

The increase of non-communicable diseases at all ages has fostered the general concern for sustaining population health worldwide. Unhealthy lifestyles and dietary habits impacting physical and psycho-social health are well known risk factors for developing life threatening diseases. Identifying the determinants of quality of life is an important task from a Public Health perspective. Consumer-Reported Outcome measures of health-related quality of life (HRQoL) are becoming increasingly necessary and relevant in the field of nutrition. However, quality of life questionnaires are seldom used in the nutrition field. We conducted a scientific literature search to find out the questionnaires used to determine the association between dietary habits and quality of life. A total of 13 studies were eligible for inclusion. Across these studies the short form-36, a generic (non-disease specific) HRQoL measurement instrument was the most widely used. However, generic measures may have limited content validity in the context of dietary habits interventions. We recommend additional contextual diet-specific HRQoL measures are also needed for evaluating the impact of diet habits on daily life functioning and well-being.

## Introduction

Although life expectancy has increased notably in the last years, non-communicable diseases at all ages are increasingly threatening the general population health globally. As people are living longer, policies and programs that enact “active aging” are a necessity. Measuring health-related quality of life (HRQoL) is related to the subjective perception of individuals’ health and well-being in relation to its social and cultural environment (Testa and Simonson, 1996). Several factors are well-known determinants of HRQoL (Corica et al., 2008; Guitérrez-Bedmar et al., 2009; Serrano-Aguilar et al., 2009). Diet, together with other aspects of daily life such as physical activity and the relation with the environment, plays a crucial role in our state of physical and mental health.

Self-perceived health status is a simple but effective indicator of overall health status and a useful tool to inform about the care needs and the organization of prevention programs (Guyatt et al., 2007). HRQoL questionnaires are an efficient way of gathering data about people’s daily functioning and psycho-social well-being. Also, health status measures have been shown to be a powerful predictor for chronic diseases and mortality over the long term in clinical practice (Wannamethee and Shapher, 1991).

It is now well established that nutrition influences outcomes in patient populations (Barr and Schumacher, 2003a,b), but few studies have assessed the relationship between HRQoL and dietary habits in the general population. Among them, the most used instrument to assess quality of life is the short form-36 (SF-36) Health Survey. However, generic health status measures may have limited content validity in the context of dietary habits interventions. So, our aim was to conduct a review to find out in the scientific literature the questionnaires used to determine the association between dietary habits and quality of life.

## Materials and Methods

The literature search was conducted in Medline, using combinations of the following terms: “diet,” “quality of life,” and “questionnaires,” including MESH-terms. In total 246 articles were selected.

The studies were evaluated applying the following inclusion criteria: (a) studies conducted exclusively in the general population (b) human studies (c) studies written in English (d) studies which used a validated assessment method and (e) studies published in the last 10 years.

**FIGURE 1:**
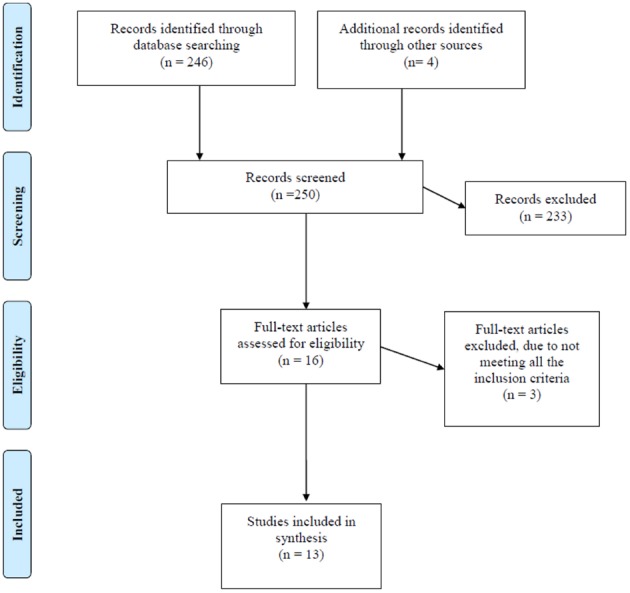
**Flow diagram of the review process**.

After initial review of titles and abstracts, 12 articles appeared to be potentially relevant, and we attempted to obtain them in full-text version. The literature lists in the selected papers were checked. We selected by handsearching four studies from this literature that met the inclusion criteria. Of these 16 potentially eligible articles, three were excluded because they did not meet all eligibility criteria. Finally 13 articles were included in this study (Figure 1).

## Results

The descriptive characteristics of the articles selected are reported in Table 1. From the 13 studies, six were cross-sectional, two randomized clinical trials, six cohort studies, and two validation studies of nutrition-specific tools for HRQoL assessment. The study samples included mostly adult populations except Costarelli et al. (2013) and Wu et al. (2012), which were conducted in young populations (10–16 years old).

**TABLE 1:** **Characteristics of included studies (*n* = 13)**.

**Authors**	**Study**	**Design**	**Country**	**Sample characteristics (group size, *n*; age, years; gender)**	**Quality of life instrument used**
Duncan et al. (2014)	10,000 Steps cohort	Cross-sectional	Australia	*n* = 10,478; ≥18 years; 70.5% women	CDC Healthy Days Instrument
Berendsen et al. (2013)	NU-Age project	RCT	European Consortium	*n* = 1,250; 65–80 years	SF-36
Ruano et al. (2013)	SUN project	Cohort	Spain	*n* = 11,128; >21 years	SF-36
Germain et al. (2013)	SU.VI.MAX trial	Cohort	France	*n* = 3,005; 45–65 years; 67.7% women	SF-36
Uritani et al. (2013)		RCT	Japan	*n* = 114; women 40–74 years	SF-36
Costarelli et al. (2013)		Cross-sectional	Greece	*n* = 359; 13–16 years	KIDSCREEN-27
Henríquez Sánchez et al. (2012)	SUN project	Cohort	Spain	*n* = 11,015; >21 years	SF-36
Wu et al. (2012)	REAL Kids Alberta	Cross-sectional	Alberta (Canada)	*n* = 3421; 10–11 years	EQ-5D-Y (youth)
Muñoz et al. (2009)		Cross-sectional	Girona (Spain)	*n* = 7,145; 25–74 years; 3,697 women	SF-12
Bonaccio et al. (2013)	Moli-Sani project	Cross-sectional	Italy	*n* = 16,937; ≥35 years; 48.4% men	SF-36
Kimura et al. (2009)		Cross-sectional	Japan	*n* = 689; ≥65 years; 401 women	QOL
Schünemann et al. (2010)		Validation study	Italy	*n* = 128; 20–65 years; 35.9% men	QUALCIBO
Guyonnet et al. (2008)		Validation study	France	*n* = 197; 20–65 years; 64% women	FBA

The majority of the studies used the SF-36 for assessing quality of life. The exceptions were the two studies conducted in adolescent populations: one study conducted by Costarelli used a specific version of a generic questionnaire for young people: EQ-5D-Y (youth), and the other study (Wu et al., 2012) used the KIDSCREEN-27 questionnaire, an instrument to assess subjective health and well-being applicable for healthy and chronically ill children and adolescents aged from 8 to 18 years. Two other studies used a specific tool for assessing HRQoL: the CDC Healthy Days instrument in the study conducted by Duncan et al. (2014), and the quantitative HRQoL for old people living in the community, in the study conducted by Kimura et al. (2009).

We identified only two nutrition-specific instruments: the Food Benefits Assessment (FBA; Guyonnet et al., 2008) and the Qualcibo questionnaire (Schünemann et al., 2010).

## Discussion

This review, designed to find out which instrument was mostly used to determine quality of life in relation to dietary habits, revealed that there are few studies that have been conducted to determine if the adherence to a specific dietary pattern could have a positive or negative influence on HRQoL in the general population. The large majority of studies regarding nutrition and quality of life have been performed in a clinical setting (Katcher et al., 2010; Cash et al., 2012) and the SF-36 was the most widely used measurement tool in this setting (Imayama et al., 2011; Villareal et al., 2011; Xu et al., 2012).

To our knowledge only two specific instruments have been developed to determine specifically the impact of diet on HRQoL in the general population, but we did not find any publication regarding their practical application, possibly because their validation is still in progress. Our results are in concordance with a recent systematic review conducted by Carson et al. (2014) regarding the effects of dietary interventions to promote weight loss on quality of life. One of the questionnaires is The FBA developed by Guyonnet et al. (2008), this questionnaire contains 41 questions that measure the impact of daily diet on eight dimensions of HRQoL, as perceived by subjects: vitality (10 items), digestive comfort (nine items), disease prevention (six items), well-being (six items), aesthetics (five items), physical appearance (three), snacking (two items), and pleasure (two items). The 41 items of the questionnaire showed good internal consistency reliability (Cronbach’s α = 0.79 to 0.91) and reproducibility. Intraclass correlation coefficient (ICC) scores exceeded the 0.70 threshold for all dimensions. When comparing FBA dimensions with SF-36 to determine the concurrent validity of the questionnaire, the Spearman correlation coefficients ranged from 0.02 (snacking) to 0.83 (well-being). No floor or ceiling effects were detected. The FBA’s sensitivity over time needs to be determined in further long-term studies, as acknowledged by the authors.

The other nutrition-specific instrument most recently developed is the Qualcibo questionnaire initially validated for Italian population by Schünemann et al. (2010). It contains 29 items across five domains to assess quality of life related to nutrition and other aspects of food intake: Healthy lifestyle (*n* = 10 items), symptoms (*n* = 6 items), sensations (*n* = 6 items), social and role function (*n* = 4 items), and taste (*n* = 3 items). The Qualcibo questionnaire still needs further longitudinal construct validity and responsiveness assessment, since the authors only performed cross-sectional validation.

The following studies illustrate that generic, disease-specific and nutrition-specific questionnaires can provide valuable insights on the impact on general quality of life, as long as the measurement tool is applicable to the context and the research question of interest.

The CDC’s Healthy Days instrument was used in a large cross-sectional on-line survey in members of the web-based 10,000 Steps project physical activity promotion initiative (www.10000steps.org.au). The study objective was to evaluate the HRQoL impact of several lifestyle behaviors, including diet (Duncan et al., 2014). The assessment of dietary behaviors included consumption of daily fruit and vegetables, soft drinks and fast foods. Poor dietary behaviors, as well as smoking, lower levels of physical activity, higher sitting time, and poor sleep behaviors, were shown to be associated with poor self-rated health and frequent unhealthy days.

In the 2001–2004 National Health and Nutrition Examination Survey, the Healthy Days questions were used to compare the impact of hypertension on general HRQoL in participants with and without hypertension (Hayes et al., 2008). A higher number of unhealthy days were reported by the respondents with hypertension.

The choice of the type of questionnaire should be based on the specific context and purpose of the study. For example, if the purpose is to compare the health status of healthy lifestyle and different diseases, the non-disease specific SF-36 is recommendable. If the purpose is to generate data that will allow to determine utilities for calculating Quality Adjusted Life Years, generic preference-based measures are recommended (e.g., EQ-5D, SF-6D, Health Utility Index; Walters and Brazier, 2003; Cruz et al., 2011).

To deepen the understanding of the impact of healthy as well as unhealthy dietary habits, we propose to start with reviewing the appropriateness of the few available nutrition-specific questionnaires for the specific purpose of your study, and search for other available measures to fill the potential contextual gaps, e.g., for measuring symptoms, (dis)satisfaction with (un)healthy diet intervention, or sleep quality.

Our study has some limitations. We limited our detailed review to those articles available in open access, so we could have missed other HRQoL instruments not available in a free format. We also limited our search to articles published in the last 10 years. We excluded articles not conducted in the general population. Given the ambiguity of the term general population, this may have led us to skip some studies in our search.

## Conclusion

From a Public Health perspective it is an important but challenging task to measure the HRQoL effects of food habits within the general population. The choice of type of questionnaires depends on the specific research question to be answered. To our knowledge only two nutrition-specific instruments have been developed to determine the impact of nutrition in the general population. Additional measurement tools are needed to explore in more depth the associations between dietary habits and their impact on population health outcomes.

## Author Contributions

CR and DD developed the study design and drafted the manuscript. LS provided guidance on analysis and data interpretation. All authors provided final approval of the manuscript.

### Conflict of Interest Statement

The authors declare that the research was conducted in the absence of any commercial or financial relationships that could be construed as a potential conflict of interest.
